# Cognitive Adaptation of Sonar Gain Control in the Bottlenose Dolphin

**DOI:** 10.1371/journal.pone.0105938

**Published:** 2014-08-25

**Authors:** Laura N. Kloepper, Adam B. Smith, Paul E. Nachtigall, John R. Buck, James A. Simmons, Aude F. Pacini

**Affiliations:** 1 Department of Neuroscience, Brown University, Providence, Rhode Island, United States of America; 2 Electrical and Computer Engineering, University of Massachusetts Dartmouth, Dartmouth, Massachusetts, United States of America; 3 Department of Zoology, University of Hawaii, Honolulu, Hawaii, United States of America; Universität Bielefeld, Germany

## Abstract

Echolocating animals adjust the transmit intensity and receive sensitivity of their sonar in order to regulate the sensation level of their echoes; this process is often termed automatic gain control. Gain control is considered not to be under the animal's cognitive control, but previous investigations studied animals ensonifying targets or hydrophone arrays at predictable distances. To test whether animals maintain gain control at a fixed level in uncertain conditions, we measured changes in signal intensity for a bottlenose dolphin (*Tursiops truncatus*) detecting a target at three target distances (2.5, 4 and 7 m) in two types of sessions: predictable and unpredictable. Predictable sessions presented the target at a constant distance; unpredictable sessions moved the target randomly between the three target positions. In the predictable sessions the dolphin demonstrated intensity distance compensation, increasing the emitted click intensity as the target distance increased. Additionally, as trials within sessions progressed, the animal adjusted its click intensity even from the first click in a click train, which is consistent with the animal expecting a target at a certain range. In the unpredictable sessions there was no significant difference of intensity with target distance until after the 7th click in a click train. Together, these results demonstrate that the bottlenose dolphin uses learning and expectation for sonar gain control.

## Introduction

Bats and toothed whales are model organisms for the investigation of sensory processing. These two animal groups convergently evolved echolocation, an active sense relying on the integration of auditory, vocal and motor systems. To forage in darkness, these animals emit intense high frequency sounds and use information from the corresponding echoes to locate, discriminate and track prey, often at great distances. Sound propagating through open space is attenuated by 6 dB for each doubling of distance to the object, and echoes returning from a small object are attenuated by a further 6 dB for each doubling of distance [1]. Assuming an ideal reflector, a target at 100 m, the detection limit of the bottlenose dolphin [2], would return an echo more than 80 dB quieter than the outgoing signal [1]. Processing such a large range of echo intensities poses a challenge for the animal's auditory system. To compensate, echolocators maintain a constant perceived echo level by changing both the transmit and receive sonar systems [3]–[8].

The biosonar imaging process determines the characteristics of a target from its echoes. A target with a fixed cross-sectional area moving to different distances requires an echolocating animal to increase its transmitted signal intensity, its receiver sensitivity, or both, to maintain a constant perceived echo strength [9]. If an animal is adapting its transmissions or receptions in this manner, any changes in the echo strength indicate changes in the target, which greatly simplifies the imaging process [3].

To adjust the auditory sensitivity to received echoes, the middle ear muscles of bats synchronously contract with each emitted echolocation signal and then relax over a short time period [3], [10]. This contraction is a protective mechanism from the loud emitted sounds, attenuating the auditory response to the bat's emitted signal; the subsequent relaxation of the muscles results in a gradual release of this attenuation. This relaxation increases the bat's hearing sensitivity over time, which combined with the signal attenuation due to transmission loss results in a constant perceived echo strength as the target distance increases [3]–[5]. Although it is still unknown whether toothed whales possess the same middle ear contraction as bats, they do possess the same middle ear anatomy as bats [11], [12] and demonstrate a similar change in hearing sensitivity according to target distance [7], [8]. This sensitivity also changes according to target characteristics [13] and may be under active control by the animal [14]. Because gain control occurs with both the transmit and receive sonar systems, the finding that the hearing sensitivity may be under active control provides motivation to investigate whether the transmit gain control is under active control as well.

Many bat [3], [15]–[22] and toothed whale species [2], [6], [23]–[26], [27] demonstrate changes in the emitted signal intensity according to target distance. Most studies estimated gain control roughly equal to the rate at which sound decreases as it propagates uniformly from a source, although these approximations may not be accurate [28]. Despite the wealth of documented changes in emitted intensity according to distance, the perceptual mechanisms behind these changes are still unknown.

The fundamental question regarding sound production and gain control in both bats and toothed whales is: are these processes fixed motor programs or are they under cognitive control? Although there are different interpretations of what constitutes “cognition,” in this paper we define cognition as the process of gathering information via the senses, creating an internal representation of the external stimulus, and acting upon that information. As such, our definition allows for experience driven modifications of the internal representation, which has already been extensively studied in other animal systems [29].

All mammals share a similar neuroanatomical organization of vocal sensory-motor integration pathways [30]. Bats' auditory and vocal motor pathways directly connect in the midbrain [31]. Despite this neuroanatomical evidence of sensory-motor integration, previous investigations into the transmit gain control of both bats and toothed whales concluded gain control “does not rely on feedback information” [17] and “is probably not the result of a cognitive process” [6]. We challenge these conclusions on the basis that prior investigations all shared one common feature: the animal was echolocating onto targets or arrays at a fixed distance, and as such, quickly developed expectations about the constant range of the target throughout the study. With such an experimental design, previous studies could not reveal whether gain control was automatic or under cognitive adjustment.

In this study, we tested the hypothesis that gain control is a fixed-motor program in the bottlenose dolphin, versus the alternative hypothesis that gain control is under cognitive control. Changes in signal intensity were measured under two different conditions of varying target distance: predictable, with constant target distance within a session, and unpredictable, with varying target distance throughout a session. Our results indicate gain control is dependent on the animal's expectation of target distance. We propose gain control is not a fixed motor program but instead is a cognitive process that relies on constant sensory feedback and experience.

## Materials and Methods

The experiments were conducted in March and June of 2013 at the floating pen complex of the Marine Mammal Research Program of the Hawaii Institute of Marine Biology off Coconut Island, Kaneohe Bay, Oahu, Hawaii. The experimental subject was a 27-year-old female bottlenose dolphin (*Tursiops truncatus*) named BJ, who measured 2.4 m and weighed 186 kg at the time of the experiment. This was a trained, experienced laboratory animal (see [32]–[35] for examples of previous experiments).

**Figure 1:**
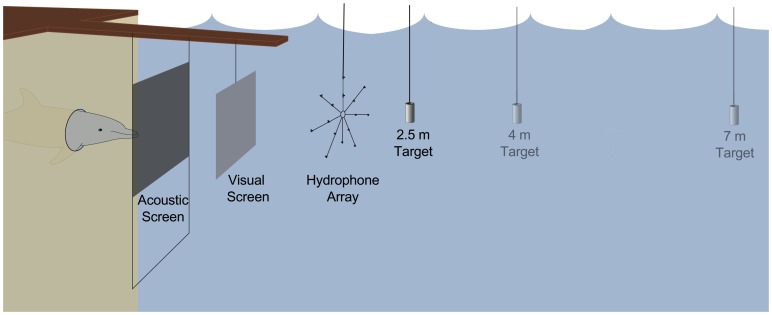
Experimental setup of the target pen. During a trial, the dolphin positioned herself into the underwater hoop 1 m below the surface of the water. An acoustically opaque metal screen was located inside the target pen, in front of the animal, to prevent her from echolocating prematurely on the targets (acoustic screen). An acoustically transparent, yet visually opaque polyethylene screen was placed in front of the acoustically opaque screen to ensure the subject did not use visual cues during target detection (visual screen). After the target was lowered 1 m into the water, the acoustically opaque screen was moved to reveal the target. The animal ensonified the target located behind the array (positioned at either 2.5, 4 or 7 m) and the hydrophone array recorded the emitted echolocation signals.

The experiment utilized two separate pens: the experimental pen, a wire enclosure measuring 8 m by 10 m that contained the dolphin; and the target pen, a wireless structure measuring 6 m by 8 m that contained the echolocation targets and the recording equipment (Figure 1). At the start of and in between trials, the subject stationed horizontally at the water surface in the experimental pen near the trainers by placing the tip of her rostrum on a vertically placed pad. When cued via a hand signal, the dolphin submerged and swam to the opposite side of the experimental pen, positioning herself into an underwater hoop up to her pectoral fins. This hoop was located 1 m below the surface of the water and allowed the animal to keep her body in the experimental pen yet position her head inside the target pen. An acoustically opaque metal screen was located inside the target pen, in front of the animal, to prevent her from echolocating prematurely on the targets. An acoustically transparent, yet visually opaque polyethylene screen was placed in front of the acoustically opaque screen to ensure the subject was not utilizing visual cues during target detection. An underwater camera (model VC-300DN, SCS Enterprises, Montebello, NY, USA) was used to monitor the dolphin's hoop behavior and ensure the animal remained stationary for the trial.

The subject was previously trained to detect the presence or absence of a hollow aluminum cylinder (the “target”) that filled with water when submerged. The target had a target strength (a measure of the reflection coefficient of the target) of −25 dB [36] and measured 12.7 cm long with an outer diameter of 37.85 mm and a wall thickness of 6.35 mm (the “standard” target [37]). After the target was lowered 1 m into the water, the acoustically opaque screen was moved to reveal the target. The subject ensonified the target pen and determined whether a target was present (a “go” trial) or absent (a “no-go” trial) using a go/no-go paradigm [38]. If the target was present, the subject backed out of the hoop and touched a response paddle with her rostrum. If the target was absent, the subject remained in the hoop until signaled out by the trainer. The subject was rewarded with fish for correct responses. Incorrect responses resulted in no fish reward.

To ensure that the animal was not cueing off acoustic or timing effects of the target lowering procedure, all trials began with a lowering of the targets into the water. For target present conditions, the target remained in the water for two seconds before the acoustic screen was lowered, and for target absent trials the target was slowly removed from the water over the two-second duration. To control for secondary cueing effects, the animal trainer was not made aware of the target condition until the animal was positioned in the hoop and facing away from the experimental shack housing the animal trainer.

A total of 6 sessions were conducted of 50 trials each: 3 sessions of unpredictable targets conducted in March and 3 sessions of predictable targets conducted in June 2013 (due to human and equipment error only 143 unpredictable trials and 142 predictable trials were recorded). All sessions contained 30 present trials and 20 absent trials. During the unpredictable sessions, the distance of the target from the dolphin's blowhole was randomized via a predetermined random permutation, with targets hung at either 2.5 m, 4 m or 7 m distance. During the predictable sessions, the distance of the target from the dolphin's blowhole was held constant for the entire session, but changed between sessions.

Echolocation signals were recorded using a hydrophone array that measured 146 cm in diameter, positioned at 1 m depth, and 2 m from the blowhole of the dolphin. The array contained 16 Reson 4013 hydrophones (Reson, Slangerup, Denmark) that were spaced approximately 25 cm apart (see Figure 1; for detail, see Figure 2 of [39]). Each hydrophone occupied an independent channel and was amplified by 20 dB using a custom-built sixteen-channel amplifier. The signals were sent to two National Instruments DAQmx-PCI 6133 analog to digital (A/D) boards (National Instruments, Austin, TX, USA) that digitized the signal of each channel at a sample rate of 1 MHz. Due to the high sampling rate and large number of hydrophones, continuous recording of the trials were unmanageable for our recording system. Instead we recorded with a circular memory buffer, triggering our system to record 1000 µs with a 200 µs pre-trigger buffer once a threshold of 20 mV was reached. This allowed each click to be recorded independently and stored for offline analysis. Prior to the experiment, the hydrophones on the array were individually calibrated using a simulated dolphin echolocation signal and demonstrated less than 1 dB variation in sensitivity. Historical temperatures for Kaneohe Bay average 22°C in March and 26°C in June [40]. Using these temperatures, assuming a signal of 130 kHz (the upper frequency of bottlenose dolphin signals), and calculating absorption with the model in [41] results in a temperature-dependent change of less than 0.005 dB for the 7 m distance between the two time periods we conducted our experiments. This change is even less for the shorter target distances.

**Figure 2:**
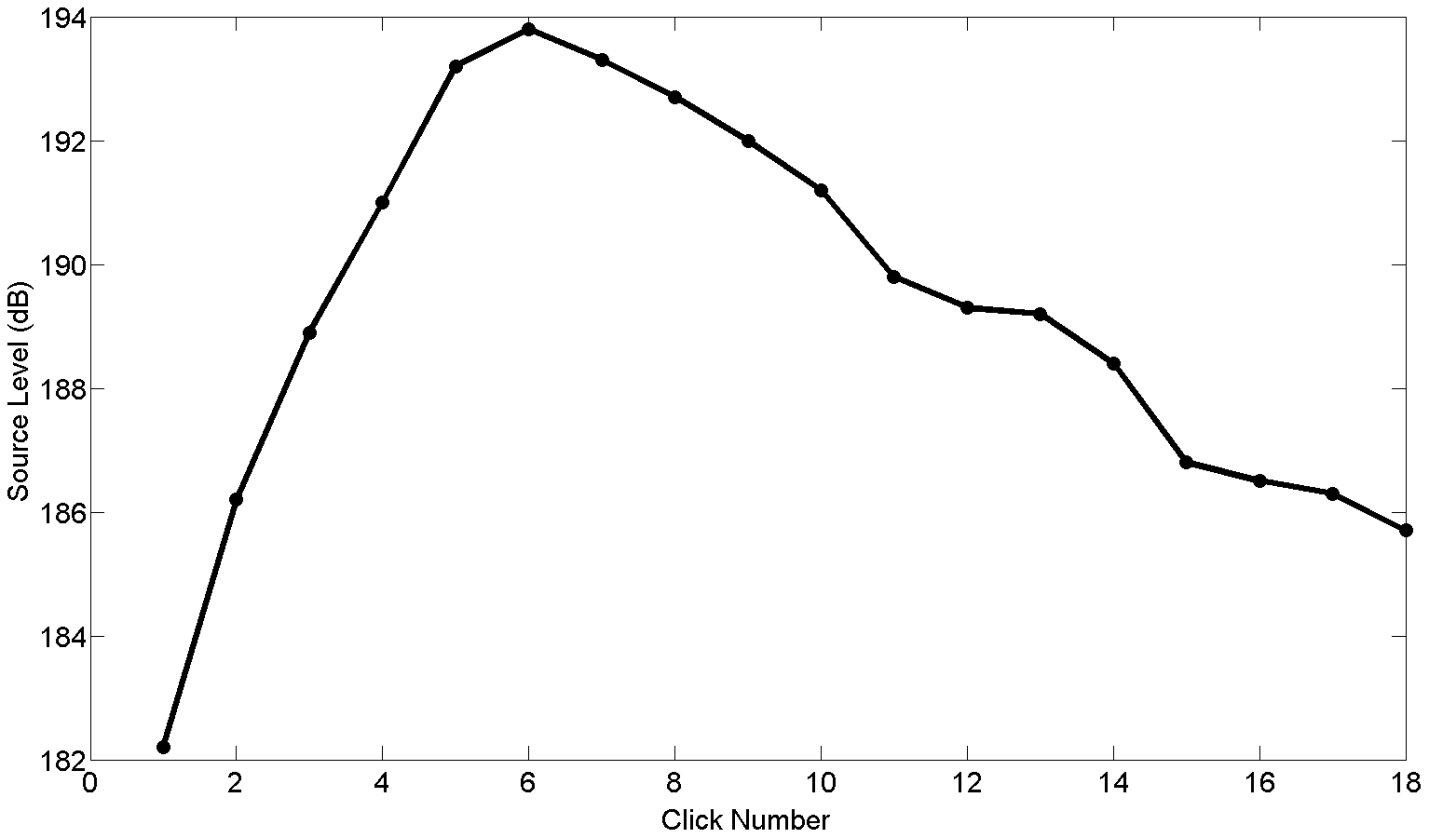
An example trial produced by the dolphin. Source levels start out around 170–180 dB, increase by 10–20 dB over a few clicks, reach maximum amplitude, and then slowly decrease. Source levels were calculated from the peak-to-peak voltage and reported in dB re: 1 µPa.

For each recorded echolocation signal, the hydrophone with the highest received sound pressure level was characterized as the on-axis hydrophone and the corresponding click recorded by that hydrophone was characterized as the on-axis click. If hydrophones on the periphery of the array were characterized as the on-axis hydrophones, the click was considered "off-axis" (because we could not rule out the possibility that the true on-axis signal was located off of the array) and eliminated from further analysis. On-axis clicks were filtered using a 12^th^ order Butterworth bandpass filter with a low frequency cutoff at 5 kHz and a high-frequency cutoff at 124 kHz. To determine the emitted click intensity for each signal, the peak-to-peak voltage was recorded and the source level (in dB re: 1 µPa) was estimated as 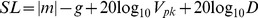
(1)where m =  the sensitivity of the hydrophone, g =  the gain (in dB) of the amplifier, V_pk_ =  the peak-to-peak voltage of each signal, and D =  the distance (in m) of the hydrophone from the dolphin's blowhole. Source levels were recorded for sequential clicks in each trial and were sorted according to target distance and target condition. The relationship between target distance and source level for each target condition was compared using a one-way ANOVA with Tukey Honestly Significant Difference (HSD) post-hoc test of significance.

Ethics Statement:

All work was approved under a National Marine Fisheries Service permit (978-15670-02) and the University of Hawaii Institutional Animal Care Committee Protocol 93-005-15. There were no field studies and no specific additional permits were required.

## Results

A total of 3,782 clicks were collected in the 143 unpredictable trials recorded in March, and 3,358 clicks were collected in the 142 predictable trials recorded in June. Throughout all of the trials the dolphin achieved a high level of echolocation performance, committing only 2 errors out of 300 trials (99.3% correct). Both of these errors were false alarms (reporting the presence of a target when the target was, however, absent) at the 2.5 m target distance for the predictable targets. The dolphin produced up to 80 clicks in each trial, with an average of 21.6±10.5 clicks per trial. Source levels of all clicks ranged from 174 to 199 dB re: 1 µPa.

The dolphin performed a generally predictable echolocation behavioral pattern for each trial regardless of session condition. Echolocation trials began with low source levels, increased rapidly in amplitude over approximately five clicks, reached a maximum source level, then declined in amplitude for the remainder of each trial (Figure 2). Due to this pattern, comparing averaged source levels across conditions would mask changes in source levels that occurred through the click train. Therefore, for each condition, clicks within a trial were indexed by click number and then the values of each click number were averaged across all trials. This preserved the time dynamics of the source levels across the trains. Due to the scanning behavior of the animal, some clicks were characterized as off-axis clicks, and not included in the analysis. This resulted in an unequal sample size for the various click numbers (Table 1). We only further analyzed click numbers at the end of the click trains that had 5 or more values.

**Table 1:** Number of data points (clicks) for each averaged click number represented in Figure 3.

	Unpredictable			Predictable		
Click Number	2.5 m	4 m	7 m	2.5 m	4 m	7 m
1	13	13	13	16	20	20
2	19	17	20	20	25	25
3	21	22	26	25	29	26
4	24	25	26	28	28	26
5	26	27	27	30	30	27
6	27	29	29	30	28	28
7	27	29	29	29	30	27
8	27	28	28	29	28	26
9	26	27	30	29	24	27
10	26	25	30	27	19	27
11	26	28	29	26	17	25
12	27	28	27	25	15	24
13	25	27	28	25	16	26
14	25	26	26	22	14	22
15	24	22	24	20	10	21
16	22	22	25	17	10	22
17	21	19	21	14	7	20
18	20	19	21	13	7	16
19	16	16	18	13	5	13
20	14	13	19	11	6	17
21	14	11	16	12	4	14
22	9	10	13	12	2	15
23	10	8	12	9	2	14
24	10	7	11	9	2	13
25	7	7	9	8	2	11
26	7	6	10	6	2	9
27	7	7	8	5	2	9
28	5	5	6	6	—	7
29	5	4	8	4	1	8
30	5	2	6	4	—	7
31	5	2	7	3	1	7
32	4	2	7	3	—	6
33	3	2	6	2	—	5
34	3	1	5	1	—	5
35	3	—	4	2	—	4
36	—	—	—	1	—	5
37	—	—	—	1	—	5
38	—	—	—	1	—	5
39	—	—	—	2	—	5
40	—	—	—	1	—	5

### Target Present Condition

**Figure 3:**
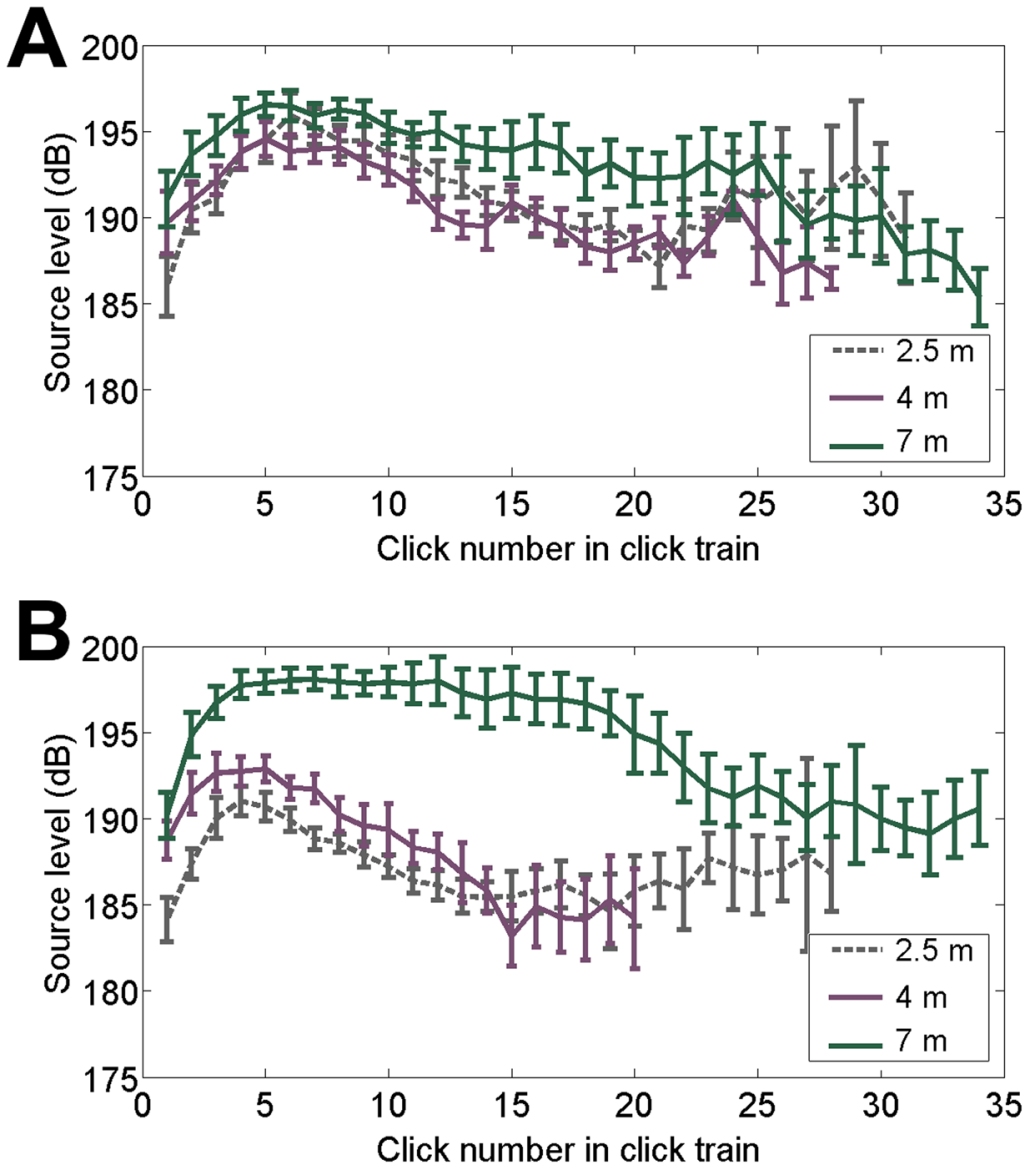
Change in source levels throughout trials according to target distance and condition. Click source levels (mean and standard error) as a function of click number for 2.5, 4 and 7 m target distance for the unpredictable targets (3a, top) and predictable targets (3b, bottom). Clicks within a trial were indexed by click number and then the values of each click number were averaged across all trials. Source levels were calculated from the peak-to-peak voltage and are reported in dB re: 1 µPa. Because some click numbers towards the end of the click train consisted of less than 5 values, we only further analyzed click numbers at the end of the click trains that had 5 or more values. See Table 1 for the number of clicks that make up each data point.

Source level patterns for the unpredictable targets did not change with target distance (Figure 3A). Trials began with source levels in the 187–191 dB range, increased to a maximum source level in the 194–196 dB range, maintained a high source level for a few clicks, then tapered back to the low −190 dB range towards the end of the trial. For the predictable targets, this pattern changed (Figure 3B). The initial source levels for the 2.5 m target is around 184 dB, much lower than those of the 4 m or 7 m distance targets. Source levels for the 2.5 and 4 m targets both increased at the same relative rate, reaching a maximum source level on the 4^th^ or 5^th^ click, although for any given click the source levels were approximately 2–3 dB higher for the 4 m target than the 2.5 m target for the first 7 clicks. After reaching a maximum, source levels for both distances decreased, returning to the 185 dB range around click 14. Source levels for the 7 m target increased at a much faster rate than the 2.5 and 4 m targets, reaching, on average, 198 dB by the 4^th^ click. Source levels began to decrease slightly but still remained in the high-190 dB range for several more clicks, returning to the low 190 dB range around click 24.

For further statistical analysis, because source levels are calculated on a logarithmic scale, it was necessary to convert source levels to their linear counterparts, voltage squared. There was a significant (*p*<0.05) effect of target distance on voltage squared for clicks 2–22 for the predictable targets, and a significant effect of target distance on voltage squared only for click 3, 8–9, 11–14, and 16–19 for the unpredictable targets (Table 2). For any of the first 14 clicks of the predictable targets, the source level increased as the target distance increased (Figure 3B). For most clicks in the predictable sessions, the source levels produced for the 7 m target distance were higher than for the 4 m or 2.5 m distance, and for most of the first 13 clicks the source levels produced for the 4 m target distance were higher than the 2.5 m distance.

**Table 2:** Summary statistics of one-way ANOVA for all the clicks produced by the dolphin for the unpredictable and predictable sessions.

Click	Unpredictable			Predictable		
	Total df	F	*p*	Total df	F	*p*
1	38	1.455	0.247	55	2.648	0.8
2	55	2.116	0.131	69	6.529	0.003
3	68	3.279	0.044	79	12.451	<0.001
4	74	2.451	0.093	81	22.393	<0.001
5	79	2.438	0.094	86	24.662	<0.001
6	83	2.33	0.1	82	22.08	<0.001
7	84	2.265	0.11	85	34.297	<0.001
8	82	4.562	0.013	82	22.263	<0.001
9	82	4.459	0.015	79	14.898	<0.001
10	80	2.446	0.093	72	12.025	<0.001
11	82	3.899	0.024	67	8.647	<0.001
12	81	7.827	0.001	63	8.25	0.001
13	79	4.251	0.018	66	9.319	<0.001
14	76	4.09	0.021	57	9.202	<0.001
15	69	2.476	0.092	50	7.902	0.001
16	68	4.116	0.021	48	7.009	0.002
17	60	3.774	0.029	40	10.889	<0.001
18	59	3.698	0.031	35	12.091	<0.001
19	49	5.208	0.009	30	10.762	<0.001
20	45	1.308	0.281	33	5.03	0.013
21	40	2.301	0.114	29	6.822	0.004
22	31	1.824	0.179	28	5.587	0.01
23	29	1.425	0.258	24	2.897	0.076
24	27	0.123	0.885	23	2.286	0.126
25	22	0.792	0.466	20	3.147	0.067
26	22	0.335	0.719	16	2.314	0.135
27	21	0.221	0.812	15	0.932	0.418
28	15	0.532	0.6	12	2.213	0.165
29	16	0.563	0.582	12	1.963	0.191
30	12	0.12	0.888	10	4.541	0.062
31	13	0.047	0.954	10	1.139	0.367
32	12	0.124	0.885	8	1.328	0.287
33	10	0.309	0.743	6	1.417	0.287
34	8	0.411	0.68	5	0.889	0.399
35	7	0.375	0.705	5	2.336	0.201
36	6	0.684	0.555	5	0.757	0.433
37	7	2.858	0.149	5	0.769	0.43
38	6	0.628	0.579	5	1.118	0.35
39	—	—	—	6	4.244	0.094
40	—	—	—	5	3.629	0.129

**Figure 4:**
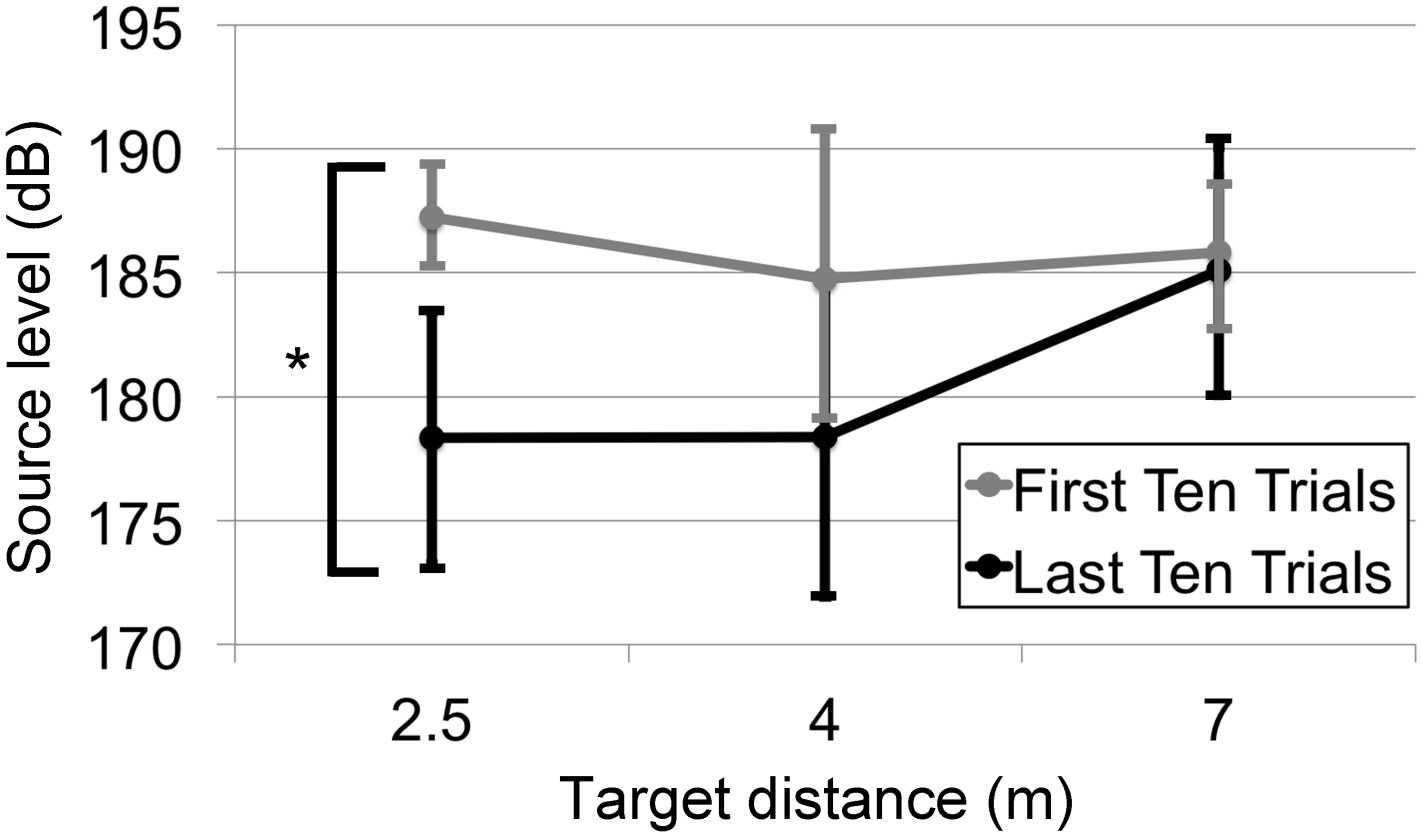
Bootstrapped 95% confidence intervals to demonstrate the effect of learning on initial click source level for the predictable trials. Bootstrapped 95% confidence intervals for the first click between the first ten trials and the last ten trials of each predictable session. Compared to the first ten trials, the dolphin produced significantly (*p*<0.05) lower source levels for the last ten trials at the 2.5 m distance. The dolphin also produced lower source levels for the last ten trials at the 4 m distance, but due to the large variation this change was not significant (*p*>0.05).

Although the ANOVA analysis for the first click of the predictable targets does not indicate a statistical difference across distances, the 2.5 m target distance has a noticeably lower initial source level than the 4 or 7 m target distance. This difference suggests a within-session learning component, which might be influenced by the predictable design of the experiment. To further investigate this time-dependent learning component, we compared the bootstrapped 95% confidence intervals [42] using the bootci function in MATLAB (Mathworks, Natick, MA) for the first click between the first ten trials and the last ten trials of each predictable session (Figure 4). In comparison to the first ten trials, the dolphin produced significantly (*p*<0.05) lower source levels for the first click of the last ten trials at the 2.5 m distance. There was no significant difference in source level between the first ten and last ten trials for the 4 m or 7 m target distance.

### Target Absent Condition

**Figure 5:**
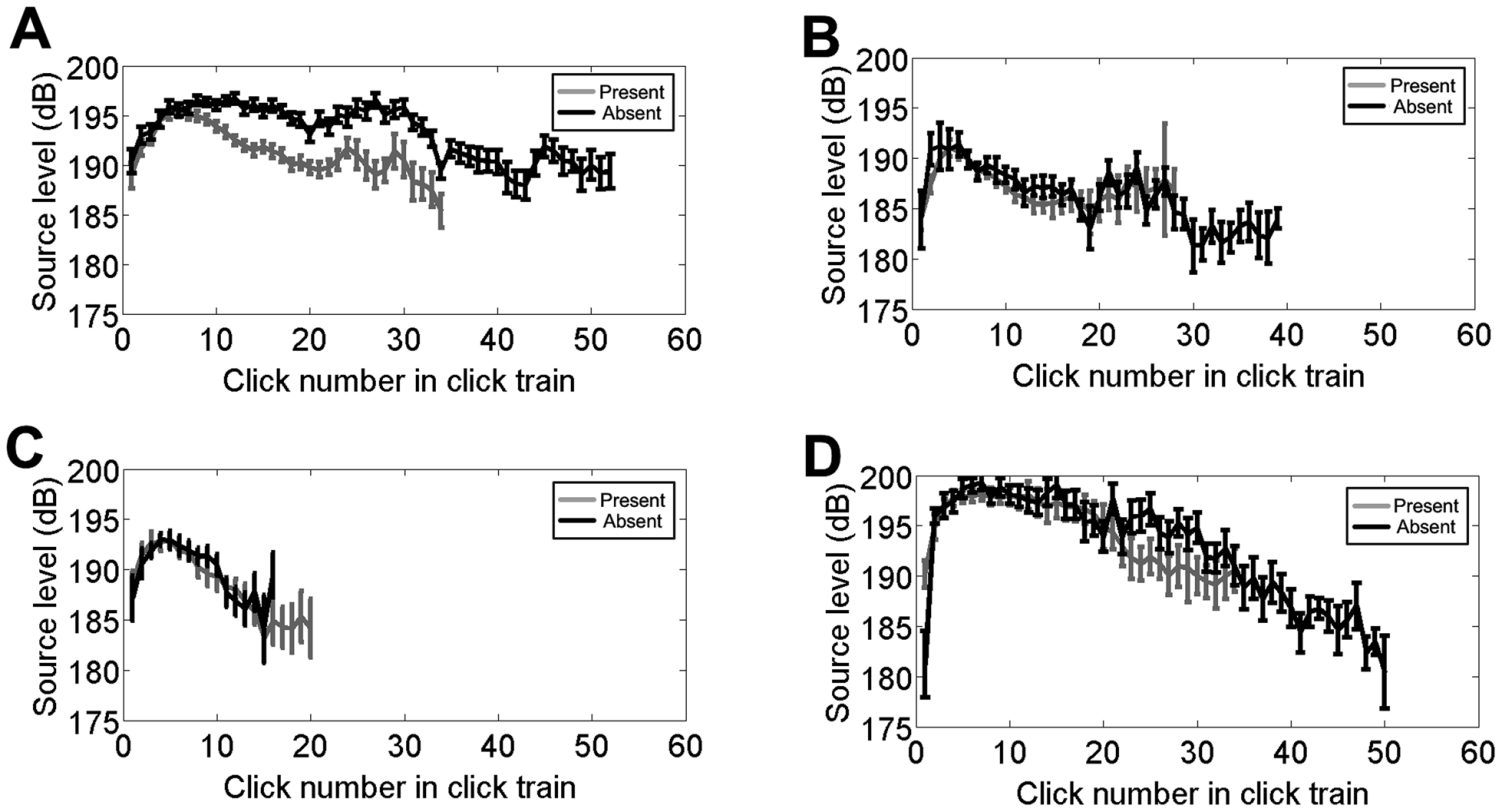
Change in source levels throughout trials according to target distance and presence. Click source levels (mean and standard error) as a function of click number for the unpredictable trials (A), and the 2.5 m (B), 4 m (C), and 7 m (D) predictable target sessions. Clicks within a trial were indexed by click number and then the values of each click number were averaged across all trials. Source levels were calculated from the peak-to-peak voltage and are reported in dB re: 1 µPa.

To investigate differences in source level output for target present versus target absent conditions, the source levels for the unpredictable targets were averaged across target distance for the target present condition and compared to the average source level for the target absent condition (Figure 5A). The dolphin produced the same source levels for the first 6–7 clicks in both the target present and target absent. For subsequent clicks, the source levels for target absent trials were maintained at a higher level than in the target present condition. For the predictable targets, there was no change in source level value or pattern between target present and target absent trials for all three target distances (Figure 5B, 5C, and 5D).

## Discussion

These results demonstrate experience is an important driver of gain control. Changes in intensity according to target distance occur only with prior knowledge of the target range. This knowledge can occur with two types of learning: within a trial and within a session. Both the predictable and unpredictable sessions are subject to within-trial learning; after receiving echoes from several clicks in the click train the dolphin learns the distance of the target and adjusts its click output accordingly. The second type of learning, within a session, is only present in the predictable sessions. The unpredictable sessions are designed to control against learning within a session.

In the predictable trials, the dolphin produced higher intensity signals as the target range increased, but in the unpredictable trials the dolphin did not produce signals with significantly different intensities according to target range until after click 7. These results provide evidence that some component of gain control is not automatic but is instead under adaptive cognitive control: dependent on target condition, learning, and expectation. One caveat is this is work derived from a single experienced animal, a common practice among working with marine mammals due to the extreme difficulty of obtaining animals for controlled laboratory conditions.

In the predictable sessions, the dolphin performed 30 trials in a row of the same target distance. If the dolphin were adapting its source level based on the target distance within a session, our hypothesis predicts distance-related changes in source level right from the first click of the later trials in a predictable session. The dolphin does indeed demonstrate such learning, decreasing the source level for the first click of the later trials of predictable sessions with the target at 2.5 m relative to the first clicks of the initial trials of the same 2.5 m sessions (Figure 4). For the farther targets at 4 m and 7 m, there is no significant change in the first click source level between the beginning and ending trials. The change in the first click source level across trials for the closest target distance explains the difference in loudness for the first click for the averaged predictable trials, as shown in Figure 3B. Because Figure 3B averages click loudness for all the trials within a session, the later trials with lower source level in the 2.5 m sessions reduce the first click average relative to the 4 m and 7 m sessions. It is also worth mentioning that for the unpredictable targets, the source level of the first click is lowest for the 2.5 m target, highest for the 7 m target, and intermediate for the 4 m target. Contrary to Figure 3B, these differences are not significant. However, simply because it cannot be excluded on a 5% level that the difference occurred by chance, it has not been shown there is no difference. Although the experimenters took every precaution to avoid secondary cueing effects, the possibility exists that the animal might be making subtle source level adjustments based on very subtle cues.

Together these data further support our cognitive control hypothesis: for predictable targets, the dolphin begins the session with the same source level (further supported by the similar values across all distances for the average source level of the first click in the first ten trials of the predictable sessions in Figure 4), but then as the session progresses the dolphin accumulates evidence as to the expected target location. This evidence then reduces the source levels for the first clicks on subsequent trials in the predictable 2.5 m session. Figure 3B also illustrates an increase in click level from one click to the next starting from click 1 - even with all trials of a predictable session pooled together. This immediate feedback of experience influencing distance-dependent source level within a click train of a predictable trial is consistent with the adaptive behavior found in other echolocating odontocetes [27].

In addition to the trial-to-trial information the animal accumulates to influence source level, the dolphin also accumulates information within a single trial's click train. If there were no within-trial accumulation of evidence, there would be no consistent change in source level across clicks of a trial for any of the target distances in the unpredictable sessions. Rather, the unpredictable trials demonstrate that, for the most part, after the 7^th^ click the dolphin does show a difference in source level, maintaining a higher intensity for the targets at 7 m distance (Table 2, Figure 3A). This suggests that once the animal gets about 8 clicks into its click train the dolphin chooses its source level based on this immediate, within-trial experience. Interestingly, for some clicks in the unpredictable sessions, the source levels produced for the 2.5 m target exceed those produced for the 4 m target, of which we cannot speculate an explanation.

The values and patterns of source levels produced for the target absent conditions (Figure 5) further support our hypothesis of cognitive gain control. For the predictable targets, the dolphin produced the same pattern of source levels regardless of whether the target was present or absent. Assuming the dolphin detects the target within the first 6–7 clicks, if the dolphin has prior expectation of the target distance and the task is to detect the presence or absence of a target at a known distance, using the same strategy for all target conditions would optimize energy output while preserving detection probability. For the unpredictable targets, the dolphin produced the same pattern and value of source levels in both the target present and target absent condition for the first 6–7 clicks, but then maintained an increased source level for the target absent condition. This indicates that for conditions of target range uncertainty, the dolphin expends more energy when it cannot detect a target within the first 6–7 clicks.

Past studies on dolphin attention demonstrate a sharp decrease in performance with unpredictable target distances [43] and, in general, information on expected target position improved detection performance [44]. Additionally, some odontocete species adjust the focus of their echolocation beam depending on target distance [45]. Therefore, if the dolphin attended to an intermediary distance, a drop in performance for the unpredictable trials would be expected. It is surprising, then, that the dolphin maintained a high level of performance for the unpredictable trials. One possible explanation is the dolphin in this study was a highly trained, experienced animal that historically performed high-resolution discrimination tasks [32]–[34]. Due to the animal's history, it may not require precise spectral or temporal features in the returning echoes for simple binary detection. For this task, even though the dolphin was adjusting its emitted intensity according to the middle expected target range, it still may have received sufficient echo information to detect the presence of the target in the water.

When the distance between an animal and its target increases, the returning echo decreases in intensity, which is calculated using the equation:
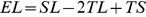
(2)where EL = echo level, SL =  source level, TL = transmission loss and TS = target strength. If the dolphin adjusted its source level to compensate for outgoing sound transmission loss, so that the incident sound has a constant level, as previously hypothesized [6], then returning echoes from a point target will decline as a function of distance only for returning propagation. However, the dolphin emitted the same intensity signals regardless of target distance for the unpredictable targets. Thus, based on Eq. 2, received echo levels for the 2.5, 4 and 7 m unpredictable target distances were 152.1, 143.9, and 134.2 dB, respectively. This represents a dynamic range for echo strength of 17.9 dB. In comparison, the received echo levels for the predictable targets calculated from the average peak source level at each distance were 147.9, 142.0, and 138.4 for the 2.5, 4 and 7 m target distances, respectively. This represents a dynamic echo range of only 9.5 dB. Because the dolphin performed the echolocation task near 100% in both conditions, this suggests the animal has an effective dynamic hearing range of at least 18 dB. Cochlear dynamic range of 35 dB is typical of most mammals [46], so even if the dolphin in this study used no gain control on either the transmit or the receive side, it could be expected to process echoes from targets at even greater distances.

If mammals have such a large dynamic hearing range, why utilize gain control in the first place? Maintaining a relatively constant perceived echo would produce auditory responses of the same magnitude as the echolocating animal approached its prey [7]. A change in echo strength would indicate a change in target orientation, suggesting evasive prey action. Constant perceived echoes allow variations in the echo strength or structure to be more readily registered by the pursuer's auditory system, which would be beneficial during pursuit of prey [3]. Therefore, gain control might be a direct adaptation to evasive strategies of prey.

**Figure 6:**
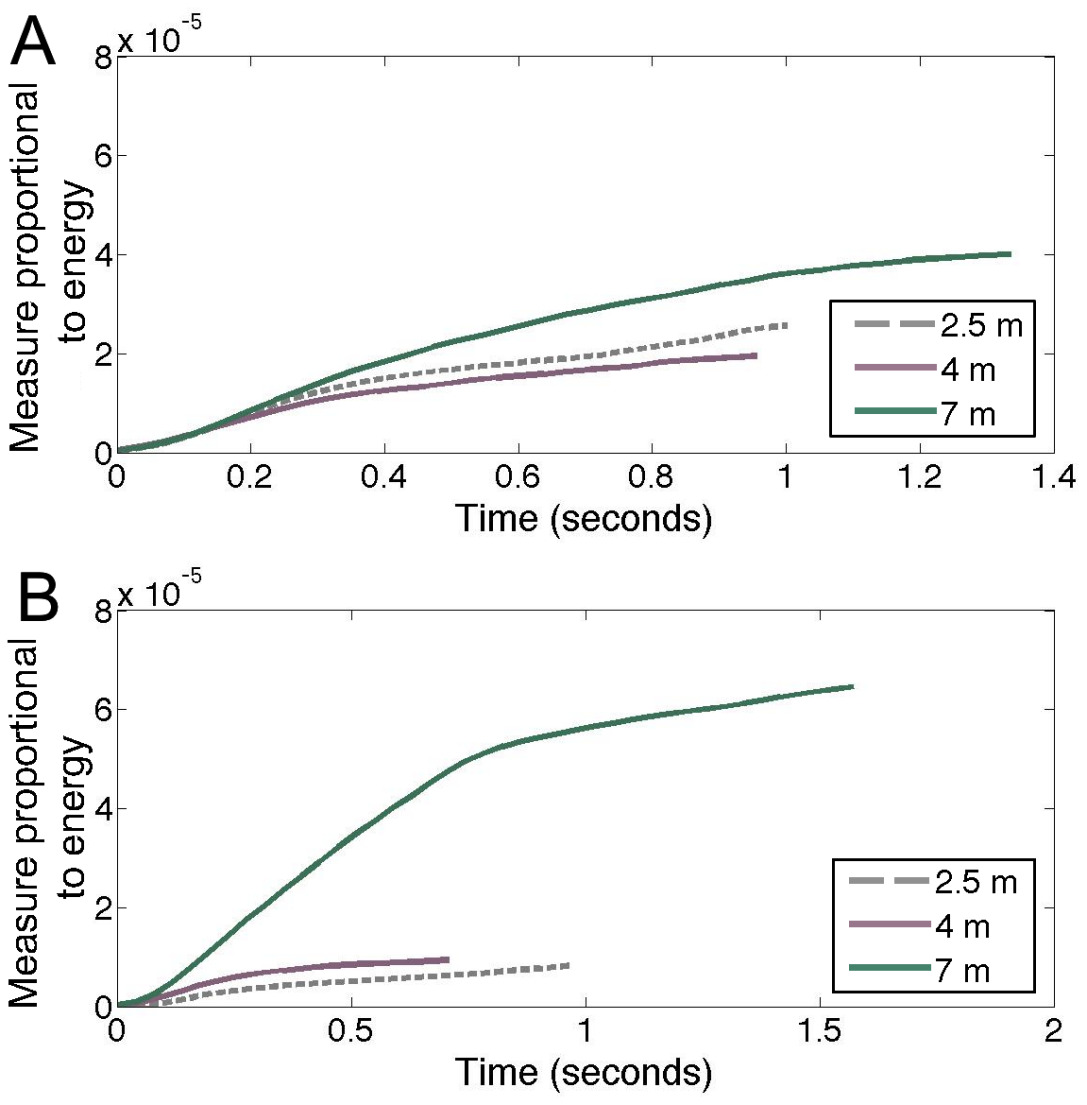
Cumulative energy over elapsed time for unpredictable targets (5a, top) and predictable targets (5b, bottom). Time was estimated based on the two-way-travel time plus a “lag” time of 30 ms. The vertical axis is (amplitude^2^ × time), which is a measure proportional to energy. This analysis further highlights the increased energy for the 7 m predictable target. For the predictable condition, the slope of the total energy versus time is much steeper than the other target distances, indicating a faster rate of source energy and therefore potential received information from the target echo.

Odontocetes typically use more than one click, or even entire click trains, to obtain information from targets [47], [48]. If odontocetes use multiple clicks, the rate at which an odontocete receives information from those clicks changes depending on target distance [49]. Because animals adapt their click rate to distance, the rate of information received from clicks depends on the target distance. As a general rule of thumb, in detection and estimation tasks increasing energy increases the amount of information available to a receiver [50]. Motivated by this, we estimated how the transmitted energy versus time varied according to target distance and condition. To investigate the rate of energy change for different target distances, the energy in one click was approximated with: 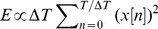
(3)in which E =  energy, ΔT = 1/sampling rate and x[n] =  discrete time signal obtained by sampling the voltage waveform in the window. Then, clicks of the same click number were averaged together for each target condition to obtain an overall average energy measurement for each subsequent click in a trial, and those values were summed across time to obtain a “running total” of average energy in each trial. This value is proportional to cumulative energy in a click train. Although the recording system used in this study did not allow for time stamps of individual clicks, odontocetes produce clicks at a rate equal to the two-way-travel time (or the time it takes for the click to travel from the dolphin to the target and back to the dolphin) plus a “lag” time of 19–45 ms, which is thought to be the time involved for neural processing or motor circuitry performance [2], [51]–[54]. Therefore, using a speed of sound of 1500 m/s, and assuming an intermediate “lag” time of 30 ms, it can be expected that the dolphin produced clicks every 33.4 ms for the 2.5 m target, every 35.4 ms for the 4 m target, and every 39.3 ms for the 7 m target. Clicks were then adjusted to a time scale based on the above reported click rate assumptions and the running total energy over the approximate elapsed time was plotted for each target condition (Figure 6). For the predictable 7 m distance target, the slope of the total energy versus time is much steeper than the other target distances, despite having fewer clicks per time. This indicates a faster rate of increase in source energy and therefore potential received information from the target echo. Therefore, the dolphin receives a much greater amount of information for the 7 m target in the predictable target sessions.

Another intriguing result from this study is that, in the predictable sessions, the animal produced the fewest number of clicks per trial for the 4 m distance. Because odontocetes produce more clicks per trial as the sonar task difficulty increases [55]–[57], we would expect the number of clicks produced per trial to increase with distance. Producing fewer clicks for the 4 m distance suggests this distance might be “easier” for the animal, although any explanation for how or why this occurs would be pure speculation.

Bottlenose dolphins are a cosmopolitan species inhabiting all oceans and seas except for polar regions. Their foraging strategies vary greatly with habitat, prey preferences and the presence of predators. The same population may even switch from individual foraging to cooperative feedings with conspecifics [58]. In cooperative feedings, individuals often have different roles in a feeding event [59], [60]. Not only would the use of a dynamic and adaptive echolocation system benefit individuals during foraging, but it would also contribute to an optimized prey intake during coordinated feeding events, particularly if animals take on different roles during the prey pursuit and capture. By adjusting their echolocation signals, animals can easily take on different roles such as herding, catching prey or preventing fish escape. These strategies are commonly observed in other species of odontocetes such as the dusky dolphin (*Lagenorhynchus obscurus*) [58] and the Hawaiian spinner dolphin (*Stenella longirostris*), which strengthens the hypothesis that gain control is present across other odontocete species.

In summary, our results support the previous hypothesis that odontocetes, in particular the bottlenose dolphin, adjust their transmitted echolocation signals according to target distance. Unlike previous studies, however, our results indicate that this adjustment is under cognitive control by the dolphin. When the dolphin has an expectation of target distance, it adjusts its click intensity according to target range, even starting with the first click of a click train. When the dolphin cannot predict the range of the target, it does not demonstrate the same source level compensation. Such a strategy of adaptive cognitive control would benefit both individuals and groups during dynamic foraging events.
